# Complete Genome Sequence of *Lelliottia nimipressuralis* Type Strain SGAir0187, Isolated from Tropical Air Collected in Singapore

**DOI:** 10.1128/genomeA.00231-18

**Published:** 2018-05-03

**Authors:** Cassie E. Heinle, Ana Carolina M. Junqueira, Akira Uchida, Rikky W. Purbojati, James N. I. Houghton, Caroline Chénard, Daniela I. Drautz-Moses, Anthony Wong, Sandra Kolundžija, Megan E. Clare, Kavita K. Kushwaha, Deepa Panicker, Alexander Putra, Nicolas E. Gaultier, Balakrishnan N. V. Premkrishnan, Vineeth Kodengil Vettath, Stephan C. Schuster

**Affiliations:** a Singapore Centre for Environmental Life Sciences Engineering, Nanyang Technological University, Singapore; b Asian School of the Environment, Nanyang Technological University, Singapore

## Abstract

Lelliottia nimipressuralis type strain SGAir0187 was isolated from tropical air samples collected in Singapore. The genome was assembled with an average coverage of 180-fold using Pacific Biosciences long reads and Illumina MiSeq paired-end reads. The genome measures 4.8 Mb and contains 4,424 protein-coding genes, 83 tRNAs, and 25 rRNAs.

## GENOME ANNOUNCEMENT

The Lelliottia genus includes environmental gammaproteobacteria, reclassified from the genus Enterobacter (1). Lelliottia nimipressuralis is a facultative anaerobic rod-shaped bacterium that has been isolated from water sources and food products and is known for its possible involvement in the wetwood disease of trees, which most notably infects elms (2, 3).

L. nimipressuralis type strain SGAir0187 was cultivated from samples taken from Singapore air using an Andersen single-stage impactor (BioStage, SKC, Inc.) and Reasoner’s 2A (R2A) agar. Samples were grown and maintained on Trypticase soy agar (TSA) (Becton, Dickinson) at room temperature and in Luria-Bertani (LB) broth overnight until they were axenic. DNA was then extracted using the Wizard genomic DNA purification kit (Promega) following the manufacturer’s instructions. The whole-genome shotgun library was then prepared for sequencing on a PacBio RS II instrument using the SMRTbell template prep kit version 1.0 and loaded onto a single-molecule real-time (SMRT) cell (Pacific Biosciences). Additional libraries were constructed using the TruSeq Nano DNA library preparation kit (Illumina) and used to generate 300-bp paired-end reads via the Illumina MiSeq platform.

A total of 80,945 PacBio subreads were assembled using the PacBio SMRT Analysis version 2.3.0 package’s Hierarchical Genome Assembly Process version 3 (HGAP3) (4), and the draft assembly was polished using the 792,437 MiSeq reads via the program Quiver (4), with error correction using Pilon version 1.16 (5). The assembly generated one chromosomal contig of 4,826,854 bp with 180.7-fold coverage and a 55.68% G+C content. This is the first report on the complete genome assembly of L. nimipressuralis.

Genome annotation was performed using NCBI’s Prokaryotic Genome Annotation Pipeline version 4.3 (6), and further functional characterization was performed via the Rapid Annotations using Subsystems Technology (RAST) server (7–9). L. nimipressuralis type strain SGAir0187 was predicted to comprise 4,424 protein-coding genes, 83 tRNA genes, 25 rRNA subunits (including 9 5S, 8 16S, and 8 23S rRNAs), 8 noncoding RNAs (ncRNAs), and 122 pseudogenes. Results from functional analysis showed 647 genes involved in carbohydrate metabolism and 483 genes involved in metabolism of aromatic amino acids and derivatives. Six adhesion genes were predicted as mediators for the hyperadherence *yidE* gene, 85 genes were found to be potentially involved in resistance to antibiotic and toxic compounds, and 14 genes were found to be involved in invasion and intercellular resistance. These genes could aid the ability of L. nimipressuralis to colonize a plant host (10).

Isolate SGAir0187 was subjected to taxon assignment methods using Phyla-AMPHORA (11) for genomic phylotyping and average nucleotide identity (ANI) analysis performed with Microbial Species Identifier (MiSI) (12). Phyla-AMPHORA showed that isolate SGAir0187 shares 94.7% identity with the genus Enterobacter, and ANI resulted in 95.92% identity with Lelliottia nimipressuralis with 0.63 confidence.

### Accession number(s).

The genome sequence of Lelliottia nimipressuralis type strain SGAir0187 was submitted to DDBJ/EMBL/GenBank under the accession number CP025034.
