# Correction for Heinle et al., “Complete Genome Sequence of Lelliottia nimipressuralis Strain SGAir0187, Isolated from Tropical Air Collected in Singapore”

**DOI:** 10.1128/MRA.00739-19

**Published:** 2019-08-08

**Authors:** Cassie E. Heinle, Ana Carolina M. Junqueira, Akira Uchida, Rikky W. Purbojati, James N. I. Houghton, Caroline Chénard, Daniela I. Drautz-Moses, Anthony Wong, Sandra Kolundžija, Megan E. Clare, Kavita K. Kushwaha, Deepa Panicker, Alexander Putra, Nicolas E. Gaultier, Balakrishnan N. V. Premkrishnan, Vineeth Kodengil Vettath, Stephan C. Schuster

**Affiliations:** a Singapore Centre for Environmental Life Sciences Engineering, Nanyang Technological University, Singapore; b Asian School of the Environment, Nanyang Technological University, Singapore

## AUTHOR CORRECTION

Volume 6, no. 18, e00231-18, 2018, https://doi.org/10.1128/genomeA.00231-18. Page 1: The title should appear as given in this correction.

Page 1, abstract, line 1: “Lelliottia nimipressuralis type strain SGAir0187” should read “Lelliottia nimipressuralis strain SGAir0187.”

Page 1, line 6: “*L. nimipressuralis* type strain SGAir0187” should read “*L. nimipressuralis* strain SGAir0187.”

Page 1, lines 25 and 26: “*L. nimipressuralis* type strain SGAir0187” should read “*L. nimipressuralis* strain SGAir0187.”

Page 2, lines 10 and 11: “Lelliottia nimipressuralis type strain SGAir0187” should read “Lelliottia nimipressuralis strain SGAir0187.”

